# Prediction of Preterm Delivery by Ultrasound Measurement of Cervical Length and Funneling Changes of the Cervix in Pregnant Women with Preterm Labor at 28-34 weeks of Gestation

**DOI:** 10.25122/jml-2020-0069

**Published:** 2020

**Authors:** Eshraghi Nooshin, Mohamadianamiri Mahdiss, Rahimi Maryam, Shafei-Nia Amineh, Noei Teymoordash Somayyeh

**Affiliations:** 1. Department of Obstetrics and Gynecology, Shahid Akbarabadi Hospital, Iran University of Medical Sciences, Tehran, Iran

**Keywords:** Cervical length, preterm delivery, funnel-shaped cervix, neonatal morbidity, neonatal mortality

## Abstract

The present study aims at predicting preterm delivery by ultrasound measurement of cervical length and the funneling changes of the cervix in preterm labor pregnant women at 28-34 weeks of gestation. The present study is an observational-analytical study with a prospective cohort design. The statistical population of this study includes 70 preterm labor pregnant women who were referred to Tehran hospitals from March 2018 to March 2020. The case group includes 35 women who had short cervical length as well as the funneling changes of the cervix. The control group includes 35 patients whose cervical length was normal and lacked the funneling changes of the cervix. The samples were analyzed after being collected. The mean age of mothers was 29.22 years in the short cervical length group (SD=4.64) and 28.45 years in the normal cervical length group (SD=4.59). The mean length of cervical length was 17.34 mm in the short cervical length group (SD=5.64) and 38.74 mm in the normal cervical length group (SD=4.53). In the case group, the delivery occurred two or seven days after the first visit; as for the proper cervical length group without funneling changes, the delivery occurred 14 days after the first delivery. Thus, the difference is statistically significant (P=0.00). In terms of the preterm delivery before week 34, there was also a significant difference between the short and normal cervical length group, as well as the groups with the funnel-shaped and non-funnel-shaped cervix (P=0.00). However, in terms of post-term delivery before week 37, there was no significant difference between short and normal cervical length groups as well as funneled and non-funneled groups (P=0.78). In terms of term labor, there was a significant difference between short and normal cervical length groups, as well as funneled and non-funneled groups (P=0.00). In investigating the cut-off point with good sensitivity, it was indicated that the cervical length and cervical funneling in pregnant women at risk predict preterm labor before week 34. With the measurement of cervical length and diagnosis of cervical funneling by applying ultrasound, preterm delivery before week 34 can be predicted. Therefore, neonatal mortality and morbidity rates can be reduced in this way.

## Introduction

Preterm delivery is one of the main health problems of the world’s health systems. It is also one of the leading morbidity and mortality factors; it accounts for two-thirds of the first-year mortality rate and half of the neonatal mortality rate in developed countries [1]. Every year, nearly 130 million babies are born globally, 4 million of whom die in the first four weeks of their neonatal life. Spontaneous preterm birth (before 37 weeks of gestation) is defined as the regular uterine contraction occurring every five or eight minutes (or less) for 30 seconds with progressive changes of the cervix. It is observed in 7-11% of pregnancies. Moreover, a genuine preterm delivery (before 34 weeks of gestation) occurs in 3-4% of pregnancies, and the likelihood of its recurrence is 6-8% [2].

Preterm delivery accounts for 75% of neonatal mortality and 50% of their nervous problems, including mental retardation, learning disorders, and cerebral palsy. These newborns not only at a high risk of the immediate complications of immaturity and the brain damages arising from hypoxic-ischemic damage, but they also suffer from the long-term complication of immaturity, including the disabilities associated with neurologic development. Moreover, these newborns are also likely to suffer from respiratory distress syndrome, hyperbilirubinemia, nutritional intolerance, Necrotizing enterocolitis, failure to thrive, increased infections, intraventricular cerebral hemorrhage, periventricular leukomalacia, retinopathy of prematurity, patent ductus arteriosus, hypotension, water-electrolyte imbalance, anemia, and hypoglycemia. In the United States, the annual cost of preterm birth has been estimated to be $26.2 billion or $51,000 per premature infant [1-6].

Given the above-mentioned issues, it is of high significance to transfer pregnant mother having preterm symptoms to level 3 medical centers equipped with a neonatal intensive care unit (NICU). It is also important to determine in which mother the preterm delivery results in preterm birth so that the patient will be transferred to NICU and avoid transferring the other cases if not required. Only by conducting a correct early diagnosis for the mothers with preterm birth can we adjust the required interventions and delay preterm birth.

Since stopping the preterm delivery process is associated with less success, today’s modern researches are now focusing on the prevention of preterm delivery. The first step to be taken for preventing preterm delivery is its prediction. In this regard, one of the main goals is the early diagnosis of women at risk and providing the required treatment in NICU. Numerous biological and biochemical factors have been applied for diagnosing and predicting spontaneous preterm delivery. Although some of these studies have provided helpful results, it must be taken into account that applying biochemical tests is both costly and time-consuming, i.e., they are not economical. One of the diagnostic tests applied for this purpose is a vaginal ultrasound that is a proper method for a morphologic evaluation of the cervix and measuring its details with high validity [7-13].

The measurement of cervical length by performing a transvaginal ultrasound is the most important marker in predicting preterm delivery. However, in most studies, an equal length has not been provided, as variable lengths (15-30 cm) have been reported [14-16]. Thus, the lack of a cut-off point is the main shortcoming and weakness of this method. Another morphologic marker of vaginal ultrasound widely used in predicting preterm delivery is internal cervical os funneling [17, 18]. Applying this marker is widely discussed; some studies have indicated a significant relationship between this marker and preterm delivery, while other studies have pointed out that there is no relationship between this marker and preterm delivery [19-21]. In some cases, despite the uterine contractions, the cervix was closed, and there was no dilatation and effacement. However, by conducting ultrasound and observing the shortened cervical length and its funneling, preterm delivery can be predicted for the following days. Therefore, it is of high significance to conduct studies for evaluating the sensitivity and specificity of these markers in this phase of pregnancy. Markers having a sensitivity for diagnosing preterm delivery before week 35 can be applied as important markers in predicting preterm delivery. Moreover, by applying this marker, the unnecessary hospitalizations, tocolysis, and inappropriate prescription of betamethasone can be prevented, and the hospital costs are reduced as well. Thus, the present study aims at predicting preterm delivery by ultrasound measurement of cervical length and cervical funneling in pregnant women with preterm labor at 28-34 weeks of gestation.

## Material and Methods

The present study is an observational-analytical study with a prospective cohort design. This study was conducted on first-time pregnant mothers (having at least one of the risk factors of preterm delivery) whose gestational age was between 28-34 weeks. The patients were referred to Tehran hospitals from March 2018 to March 2020. Written informed consent was obtained from all patients.

Given the 5-10% rate of preterm delivery among pregnant women, the confidence interval of 95% (α= 0.05), and accuracy of 3% (p=0.03), the required sample size of the present study was determined to be 70 participants. All first-time, singleton women were investigated with the complaint of preterm delivery with gestational age between 28-34 weeks. They had about four contractions in 20 minutes. The investigation continued so that as many as 70 samples were studied from each group (35 samples with short cervical length and funneling and 35 samples with normal cervical length).

All examinations were conducted by the same researcher. The patients were included in the present study based on the inclusion and exclusion criteria described below, being required to complete the designed questionnaires. Moreover, the participants underwent transvaginal ultrasound for the measurement of cervical length and funneling changes of the cervix. They were also followed-up until their delivery, and the final questionnaire was completed as well. Ultrasound examination was conducted with a Philips machine and a vaginal probe using the standard method. Pregnant women emptied their bladder before the ultrasound. They were placed in a dorsal lithotomy position; when a standard image was acquired, the probe was taken out. The cervical length refers to the distance between the internal and external cervical os (the length of the lower end of the uterus). Cervical funneling occurs when the opening of the internal cervical os changes into a U-shape or a V-shape and its width is larger than 5 mm.

The inclusion criteria were the following: 18-to-40-year-old women, first-time pregnancy, singleton pregnancy, gestational age of 28-34 weeks, visiting the hospital with the symptoms and complaints of preterm labor, having healthy membranes, consent to research.

Exclusion criteria included multiple pregnancies, the presence of fetal anomalies, amniotic sac rupture, preterm premature rupture of membranes (PPROM), mothers with underlying diseases, presence of a cerclage thread, suspected placenta previa, gestational age of less than 28 weeks and more than or equal to 34 weeks, severe vaginal bleeding, the presence of symptoms related to issues other than idiopathic spontaneous labor such as trauma.

### Data collection and data collection tools

The data of the present study were collected by using questionnaires, and tocodynamometers were used to confirm the patient’s preterm labor. Patients with the possibility of preterm delivery underwent necessary treatments. A follow-up procedure was conducted until delivery. The patients’ data were recorded on the checklists.

### Ethical considerations

The patients’ information remained confidential. The patients’ names were not used in the present study; we used special codes for each patient. Written letters of consent were obtained from the patients before entering the present study. The patients were not required to pay for ultrasound examination. No aggressive or risky action was conducted against maternal or fetal health. Moreover, there was no financial benefit, either directly or indirectly, for the researchers in the present study. The results of the present study are not applied for making medical decisions, and there is no financial benefit for the patients either.

### Data analysis

The collected data were analyzed by using SPSS, version 16. The Chi-squared test and Fisher’s Exact Tests were applied as well. ROC curve was used for determining the sensitivity and specificity. The significance level was determined to be less than 0.05. In applying the ROC curve, for the maximum diagnostic accuracy (sensitivity of 100%, specificity of 100%), the level was required to be under curve 1, and for the least accuracy (where the diagnosis is completely based on chance), the level was required to be under 0.5.

## Results

Seventy pregnant women whose gestational age was 24-38 weeks underwent vaginal ultrasounds. The mean age of mothers was 29.22 years in the short cervical length group (SD=4.64) and 28.45 years in the normal cervical length group (SD=4.59). The difference was not statistically significant (PV=0.99). The mean pregnancy age was 31.08 years in the short cervical length group (SD=1.75) and 30.68 years in the normal cervical length group (SD=1.76); the difference was not statistically significant (PV=0.53). The mean cervical length was 17.34 mm in the short cervical length group (SD=5.64) and 38.74 mm in the normal cervical length group (SD=4.53); the difference was statistically significant (PV=0.04). In the short cervical length group, 26 cases (74.3%) used Cyclogest®, while only 5 cases (SD=14.3%) of the normal cervical length group used it; the difference was statistically significant (PV=0.00).

In the short cervical length group, as many as 7 cases (20%) underwent delivery two days after visiting the hospital, while in the normal cervical group, 0 cases (0%) underwent delivery two days after visiting the hospital; this difference was statistically significant (PV=0.00). In the short cervical length group, 13 cases (37.1%) underwent delivery seven days after visiting the hospital, while in the normal cervical group, and 0 cases (0%) underwent delivery seven days after visiting the hospital; this difference was statistically significant (PV=0.00). In the short cervical length group, 25 cases (71.4%) underwent delivery within fourteen days after visiting the hospital, while in the normal cervical group, 1 case (2.9%) underwent delivery within fourteen days after visiting the hospital; this difference was statistically significant (PV=0.00). Finally, in the short cervical length group, 10 cases (28.4%) underwent delivery fourteen days after visiting the hospital, while in the normal cervical group, 34 cases (79.1%) underwent delivery fourteen days after visiting the hospital; this difference was statistically significant (PV=0.00) (Table 1).

**Table 1:** Delivery difference according to visiting times between the groups (short and normal cervical lengths and funnel-shaped and non-funnel-shaped cervix).

Delivery time	Short cervical length group	Normal cervical length group	P-Value
	N (%)	N (%)	
**Delivery two days after visiting the hospital**	7 (20%)	0 (0%)	0.00
**Delivery seven days after visiting the hospital**	13 (37.1%)	0 (0%)	0.00
**Delivery within fourteen days after visiting the hospital**	25 (71.4%)	1 (2.9%)	0.00
**Delivery fourteen days after visiting the hospital**	10 (28.6%)	34 (79.1%)	0.00
**Delivery time**	Funnel shaped cervix group	Non-funnel-shaped cervix group	P-Value
**Delivery two days after visiting the hospital**	6 (50%)	1 (1.7%)	0.00
**Delivery seven days after visiting the hospital**	12 (100%)	1 (1.7%)	0.00
**Delivery within fourteen days after visiting the hospital**	12 (100%)	14 (24.1%)	0.00
**Delivery fourteen days after visiting the hospital**	12 (100%)	44 (75.9%)	0.00

In the short cervical length group, 26 cases (74.3%) underwent preterm premature labor before week 34, while in the normal cervical length group, only 1 patient (2.9%) underwent preterm labor before week 34; this difference is statistically significant (PV=0.00). In the short cervical length group, 9 cases (25.7%) underwent preterm premature labor before week 37, while in the normal cervical length group, 8 patients (22.9%) underwent preterm premature labor before week 37; this difference is not statistically significant (PV=0.78). In the short cervical length group, no case (0.0%) underwent term labor, while in the normal cervical length group, 24 patients (74.3%) underwent term labor; this difference is statistically significant (PV=0.00) (Table 2).

**Table 2:** The labor difference at preterm times in the two groups (short vs normal cervical length and funnel-shaped vs. non-funnel-shaped cervix).

Labor time	Short cervical length group	Normal cervical length group	P-Value
	N (%)	N (%)	
**Preterm premature labor before week 34**	26 (74.3%)	1 (2.9%)	0.00
**Preterm premature labor before week 37**	9 (25.7%)	8 (22.9%)	0.78
**Term labor**	0 (0%)	26 (74.3)	0.00
**Labor time**	**Funnel-shaped cervix**	**Non-funnel-shaped cervix**	**P-Value**
**Preterm premature labor before week 34**	10 (83.3%)	17 (29.3%)	0.00
**Preterm premature labor before week 37**	2 (16.7%)	15 (25.9%)	0.49
**Term labor**	0 (0%)	26 (44.8%)	0.00

From the 12 cases of the funneling cervix group, 6 cases (50%) underwent delivery 2 days after visiting the hospital, and from the 58 cases of the non-funneling cervix, only 1 case (1.7%) underwent delivery two days after visiting the hospital; this difference is statistically significant (PV=0.00). From the 12 cases of the funneling cervix group, 12 cases (100%) underwent delivery within 7 days after visiting the hospital, and from the 58 cases of the non-funneling cervix, only 1 case (1.7%) underwent delivery within 7 days after visiting the hospital; this difference is statistically significant (PV=0.00). Among the 12 cases of the funneling cervix group, 12 cases (100%) underwent delivery within 14 days after visiting the hospital, and among the 58 cases of the non-funneling cervix, 14 cases (24.1%) underwent delivery within 14 days after visiting the hospital; this difference is statistically significant (PV=0.00). From the 12 cases of the funneling cervix group, 12 cases (100%) underwent delivery 14 days after visiting the hospital, and from the 58 cases of the non-funneling cervix, 44 cases (75.9%) underwent delivery 14 days after visiting the hospital; this difference is statistically significant (PV=0.00) (Table 1).

Of the 12 cases of funneling cervix, 12 cases (100%) underwent delivery within 14 days after visiting the hospital, while of the 58 cases with the non-funneling cervix, only 14 cases (24.1%) underwent delivery within 14 days after visiting the hospital; this difference is statistically significant (PV=0.00).

From the 12 cases of the funneling cervix group, 10 cases (83.3%) underwent preterm premature labor before week 34, and from the 58 cases of the non-funneling cervix group, 17 cases (29.3%) underwent preterm premature labor before week 34; the difference is statistically significant (PV=0.00). From the 12 cases of the funneling cervix group, only 2 cases (16.7%) underwent preterm postmature labor before week 37, and from the 58 cases of the non-funneling cervix, 15 cases (25.9%) underwent preterm postmature labor before week 37; the difference is not statistically significant (PV=0.49). From the 12 cases of the funneling cervix group, no case (0%) underwent term labor, and from the 58 cases of the non-funneling cervix, 26 cases (44.8%) underwent term labor; the difference is not statistically significant (PV=0.00) (Table 2).

**Figure 1:**
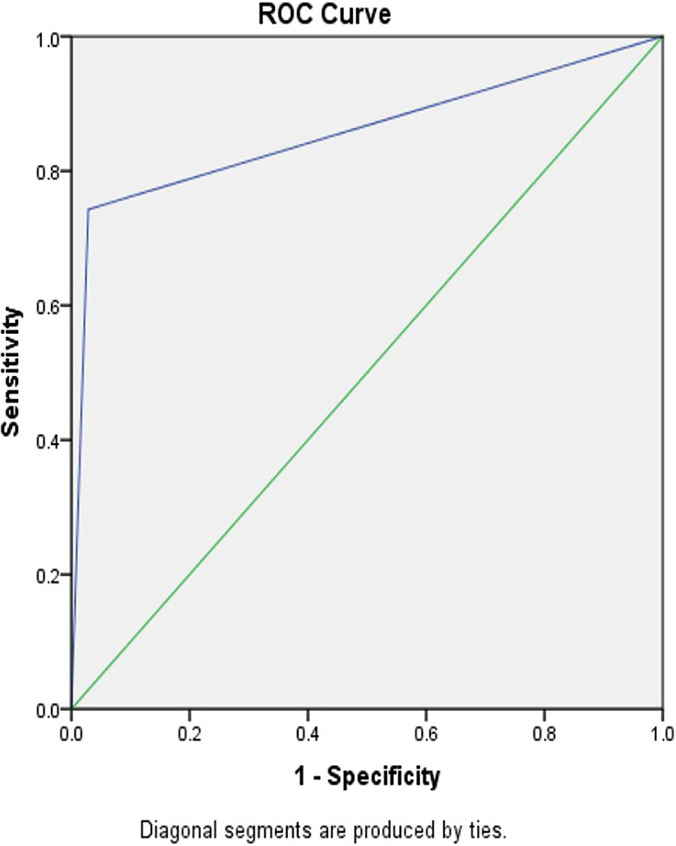
Sensitivity and specificity of funneling cervix and preterm labor.

**Figure 2:**
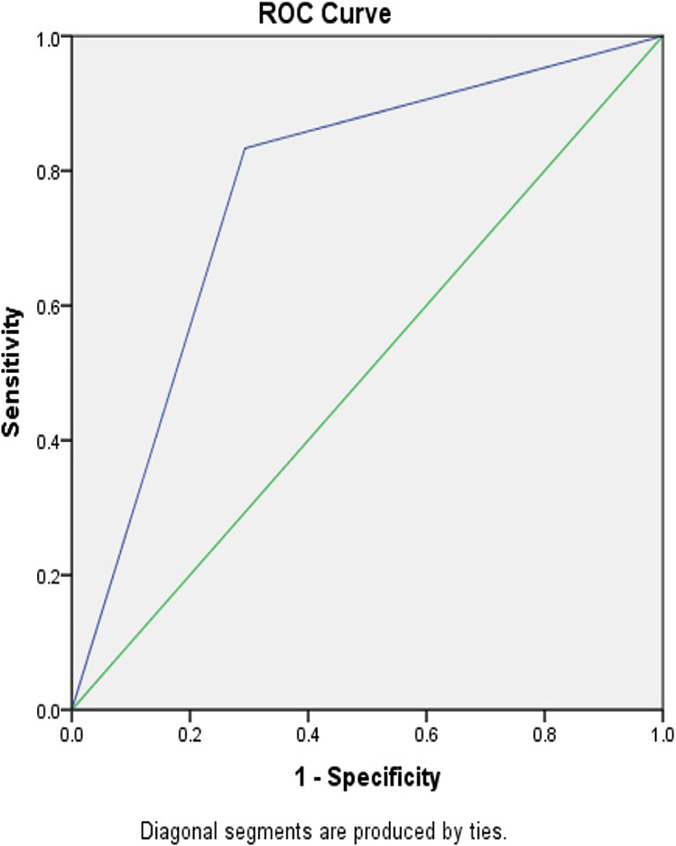
Sensitivity and specificity of cervical length and preterm labor.

The results of logistic regression analysis based on the cervical length and its relationship with preterm labor indicated that a cervical length of less than 25 mm increases the likelihood of preterm labor before week 34 (in comparison to women having a cervical length of more than 25 mm). In the same way, the funneling cervix increased the likelihood of preterm labor before week 34 (in comparison to women without a funneling cervix) (Table 3, Figures 1 and 2).

**Table 3:** Statistical results of ultrasound markers and their relationship with preterm labor before week 34.

Ultrasound markers	Rank	Number	Sensitivity	Specificity	P	Crude OR
Cervical length	Shorter than 25 mm	35	0.74	0.02	0.000	0.2
	**Normal**	35				
Funneling cervix	Funnel-shaped	12	0.83	0.29	0.000	0.2
	**Non-funnel shaped**	35				

## Discussion

The diagnosis of preterm labor is of the main purposes of reducing neonatal mortality. Preventing preterm labor depends on recognizing the main mechanisms of preterm labor. In this regard, applying screening methods seems necessary for determining risky and preterm pregnancies. Different studies have recommended conducting cervical ultrasounds instead of manual examinations [22]. Vaginal ultrasound can provide an accurate, objective, and repeatable measurement for determining the cervical length and observing whether the cervix is funnel-shaped or not [22, 23]. It seems highly important to apply tools for diagnosing preterm labor, tools that have the required sensitivity and specificity for differentiating low-risk women from high-risk ones [22, 24] because it results in the reduced hospital stay. These hospitalizations commonly lead to prescribing tocolytic treatments whose potential harmful effects are widely suspected. Moreover, such measures bring about various forms of stress and costs for pregnant women and their families [9-13].

Effacement or cervical shortness are associated with an increased risk of preterm labor. For many years, the cervical length used to be measured with the fingers; this method was commonly associated with measurement errors. However, in recent years, ultrasound has offered a new method for evaluating cervical length [26, 27]. The results of the present study indicated that the cervical length of less than 25 mm increases is associated with preterm labor before week 34. Moreover, the results of the present study indicate that the cervical length of less than 25 mm, and the funneling shape of the cervix can predict preterm labor. Different studies have reported different cut-off points of cervical length for predicting preterm labor [23, 25-29]. In the present study, based on the ROC curve, the cut-off point of 25 mm had the highest sensitivity and specificity for predicting preterm labor before week 34. The results of other studies indicated that the cervical length of less than 20 mm could predict preterm labor before week 34 and 37. The main cause of different cervical lengths and cut-off points in other studies is possibly the participation of women with different gestational ages as well as women having more than one labor; it has been confirmed in different studies that the cervical length in multiparous pregnant women is larger than that of nulliparous women.

The results of the present study indicated that 12 patients (17%) of the entire population had funnel-shaped cervices. Moreover, the results of the present study indicated that the funneling of the cervix did not have a significant relationship with preterm labor before week 37. However, there was a significant relationship between the funneling of the cervix and preterm labor before week 34. Different studies have confirmed the relationship between the funneling of the cervix and preterm labor before week 35. However, no significant relationship has been observed between the funneling of the cervix and preterm labor before week 37 [27-33, 29-35]. Thus, it can be concluded that the funneling of the cervix is a weak marker for predicting preterm labor earlier than week 37.

## Conclusion

The measurement of cervical length by performing a vaginal ultrasound can predict preterm labor before week 34 in pregnant women at risk. Thus, neonatal morbidity and mortality rates are reduced. Moreover, with the measurement of cervical length, the prescription of potentially dangerous drugs and unnecessary hospitalization can be prevented for women mistakenly diagnosed with preterm labor.

## Conflict of Interest

The authors declare that there is no conflict of interest.
